# HER2 Expression in Fine Needle Aspirates of Lymph Nodes Detected by Preoperative Axillary Ultrasound in Breast Cancer Patients

**DOI:** 10.1371/journal.pone.0113065

**Published:** 2014-11-13

**Authors:** Ji Soo Choi, Hyun Ok Kim, Eun-Kyung Kim, Young Joo Suh, Jung Hyun Yoon, Hee Jung Moon, Min Jung Kim

**Affiliations:** 1 Department of Radiology, Research Institute of Radiological Science, Yonsei University College of Medicine, Seoul, Korea; 2 Department of Radiology, Samsung Medical Center, Seoul, Korea; 3 Department of Laboratory Medicine, Yonsei University College of Medicine, Seoul, Korea; Penn State Hershey Cancer Institute, United States of America

## Abstract

The purpose of this study was to assess the usefulness of HER2 levels in ultrasonographically guided fine-needle aspiration biopsy (US-FNA) aspirates of axillary lymph nodes (ALNs) in the determination of lymph node metastasis or the characterization of primary breast cancer, and to correlate the HER2 levels in US-FNA aspirates (FNA-HER2s) of metastatic ALNs with the HER2 statuses of corresponding primary breast cancers. An institutional review board approved the study. Between January and October 2010, 164 patients with 167 ALNs examined by US-FNA were included. FNA-HER2s of ALNs were measured by chemiluminescence immunoassay, and they were correlated with cytologic/final diagnoses. Receiver operating characteristics (ROC) curve analysis was performed to evaluate the diagnostic ability to differentiate benign and metastatic ALNs. Additionally, FNA-HER2s of metastatic ALNs were correlated with HER2 status and other clinicopathologic variables of the primary breast cancers. Among the 167 ALNs, 138 were metastatic and 29 were benign. The mean FNA-HER2 (6.3 ng/ml) of metastatic ALNs was higher than that of benign ALNs. All 29 benign ALNs showed no measurable value of FNA-HER2 (0.0 ng/ml). The area under the ROC curves of FNA-HER2 of ALNs was 0.679 for the diagnosis of ALN metastasis. The FNA-HER2 statuses of 108 metastatic ALNs (79.4%) were concordant with the HER2 statuses of the corresponding primary breast cancers. In a subgroup analysis of HER2-positive cancers with ALN metastasis, distant metastasis was significantly associated with FNA-HER2-negativity of metastatic ALNs (*P* = 0.04). Although FNA-HER2 of ALNs did not improve the diagnostic performance of FNA cytology in preoperative diagnosis of ALN metastasis of overall patients, FNA-HER2-positive metastatic ALNs were significantly associated with HER2-positivity of primary breast cancers. Additionally, FNA-HER2 analysis of ALN may help to develop more personalized treatment protocol for breast cancer patients by determining the concordance or discordance of HER2 status between primary cancers and metastatic ALNs.

## Introduction

Preoperative evaluation of axillary lymph node (ALN) status is important to predict prognosis and to decide treatment plans for breast cancer patients [1]–[3]. Axillary ultrasound (US) and adjunctive US-guided fine needle aspiration biopsy (US-FNA) for ultrasonographically suspicious ALNs are widely used for preoperative staging of ALN, because of their simplicity and high specificity, ranging up to 100% [3]–[7]. Although sentinel lymph node biopsy (SLNB) is a standard procedure of ALN evaluation in patients with invasive breast cancer [8], [9], patients who have positive cytologic results on preoperative US-FNA directly undergo axillary lymph node dissection (ALND) or neoadjuvant chemotherapy (NAC). However, broad ranges for the sensitivities (21 to 86%) and negative predictive values (65 to 90%) of axillary US combined with US-FNA have been reported [3]–[7], [10], [11], which means that axillary US combined with US-FNA may lead to a substantial number of false-negative cases in certain circumstances. Consequently, SLNB is performed to confirm ALN status for patients who have invasive breast cancers and negative or indeterminate results on axillary US combined with US-FNA, even though SLNB is an invasive surgical procedure with a risk of radiation exposure to the operating team, and has associated costs and a prolonged operation time [11]. Therefore, non-surgical accurate methods that can preoperatively determine ALN status would be clinically beneficial to simplify the ALN staging process and to reduce unnecessary SLNB. Given this situation, several studies have focused on identifying tumor markers to determine ALN metastasis using fine needle aspirates of ALN. Carcinoembryonic antigen (CEA), breast cancer antigen 15-3 (CA 15-3), and cytokeratin fragment 21-1 (CYFRA21-1) have been proposed to be tumor markers which may be helpful for preoperative diagnosis of ALN metastasis in breast cancer patients [12], [13].

Metastatic cells obtained though needle-aspiration from axillary lymph nodes can reveal characteristics with phenotypes of breast cancer. One representative subtype of breast cancer is human epidermal growth factor receptor 2 (HER2) type. HER2 is an oncogene located on chromosome 17q21 and encodes a transmembrane glycoprotein with tyrosine kinase activity involved in regulation of cell growth [14]. Amplification or overexpression of the HER2 gene is identified in 15–30% of breast cancers and is associated with more aggressive disease and poor prognosis [15]. The HER2 receptor can be a target for trastuzumab, which has proven to be an effective treatment for HER2-positive breast cancer [16]. Therefore, HER2 status evaluation is vital in the pretreatment planning of therapeutic strategies. Immunohistochemical (IHC) and fluorescence in situ hybridization (FISH) analyses using formalin-fixed, paraffin-embedded tissue samples are the most widely used methods for measuring HER2. However, in some cases of metastatic or recurrent disease, only fine needle aspiration biopsy (FNA) specimens might be available. In these circumstances, it would be helpful to be able to identify HER2 status using FNA specimens.

The HER2 receptor consists of a cytoplasmic domain with tyrosine kinase activity, a transmembrane domain, and an extracellular domain (ECD). The ECD of the HER2 receptor can be cleaved and released from the surface of breast cancer cells, and can be detected by enzyme-linked immunosorbent assay (ELISA) [17]. Using this method on serum samples, many researchers have reported that higher serum HER2 concentrations are associated with decreased treatment response and poor prognosis [18]–[20]. Therefore, we hypothesized that it might be possible to determine the HER2 status of FNA specimens from ALNs via ELISA, and that this might have clinical utility in breast cancer treatment.

The purpose of this study was to assess the usefulness of HER2 level measurement of US-FNA specimens (FNA-HER2) from ALNs in the determination of lymph node metastasis or characterization of primary breast cancer, and to compare the HER2 status of metastatic ALNs based on FNA-HER2 with that of primary breast cancer tissue.

## Methods

### Study population

This study was approved by the institutional review board of Severance Hospital, Yonsei University College of Medicine, and written informed consent was obtained from all patients. Between January and October 2010, 167 women with invasive breast cancers underwent US-FNA of 170 ALNs at our institution. Among these, three patients who had previous mastectomies due to malignancy were excluded. Ultimately, 164 patients with 167 ALNs examined by US-FNA were included in this study. One patient had bilateral cancer and underwent US-FNA of bilateral ALNs, and two patients underwent US-FNA of ipsilateral and contralateral ALNs.

### US and US-FNA of axillary lymph nodes

Preoperative axillary US was performed by seven breast radiologists with various degrees of experience (2 to 13 years). US equipment included Phillips ATL HDI, iU22 (Phillips-Advanced Technology Laboratories, Bothell, WA, USA) machines equipped with high-frequency linear transducers (5 to 12 MHz). Bilateral axillary areas were scanned in an orthogonal direction along the axillary artery from the lower axilla to the junction of the axilla and upper arm. The following US features were used to identify metastatic ALNs: loss of fatty hilum, cortical thickening (>3 mm), irregular or round shape, markedly hypoechoic cortex, or increased peripheral blood flow on Doppler US [13], [21], [22]. ALNs were considered suspicious when one or more suspicious US features were detected, and US-FNA was recommended for all suspected metastatic ALNs.

US-FNA was performed with a 23-gauge needle attached to a 2-ml disposable syringe using freehand technique. Each lymph node was aspirated at least twice. Immediately after aspiration, samples were expelled onto glass slides and fixed in 95% alcohol for Papanicolaou staining. The remnants in the needle and syringe were rinsed with 1 ml of normal saline, and then the washout was made. Each washout was centrifuged at 1,200 revolutions per minute for 5 min at 4°C. The supernatants were carefully collected for HER2 assays.

### FNA-HER2 analysis

HER2 levels in FNA specimens (FNA-HER2) of ALNs were measured by chemiluminescence immunoassay (ADIVIA Centaur System, Siemens, Tarrytown, NY, USA), which is a two-site sandwich immunoassay. Reagents contained two monoclonal antibodies specific for unique epitopes on the extracellular domain of the HER2 receptor.

### Pathologic analysis

The cytologic results of FNA specimens were interpreted by five cytopathologists with 1 to 15 years of experience, and were divided into two categories. Reports of metastasis from breast cancer or atypical cells were considered positive. Reports of reactive hyperplasia or specific benign diagnoses (e.g. tuberculosis) were considered negative. Reports of insufficient material were considered negative, because a patient with insufficient material on cytologic report should undergo SLNB just like a patient with negative cytologic results [4], [13], [23].

All 165 primary breast cancers were confirmed by preoperative core needle biopsy. Among the 164 patients, 144 patients (87.8%) had breast surgery and subsequent SLNB or axillary lymph node dissection: unilateral breast surgery and ipsilateral SLNB/ALND (n = 143), or unilateral breast surgery and bilateral ALND due to contralateral ALN metastasis (n = 1). Among these patients, 102 received NAC before surgery. Twenty patients (12.2%) had not been operated on due to presence of distant metastasis. Among these, one patient had bilateral breast cancer and bilateral metastatic ALNs, and one patient had unilateral breast cancer and bilateral metastatic ALNs. For primary breast cancers, final histopathologic results of surgical or core needle biopsy specimens were used as reference standards. For ALNs, surgical histopathologic results (n = 145) or the combination of cytologic results and clinical evidence during follow-up (n = 22) were used as reference standards. Among the ALNs confirmed by surgery, the aspirated ALNs were correlated by the location seen at surgery.

IHC staining for HER2, estrogen receptor (ER), progesterone receptor (PR), androgen receptor (AR), and Ki-67 were performed on tissue blocks. Briefly, 5-µm sections were obtained with a microtome, transferred onto adhesive slides, and dried at 62°C for 30 minutes. After incubation with primary antibodies against HER2 (polyclonal, 1∶1, 500; DAKO, Glostrup, Denmark), ER (clone SP1, 1∶100; Thermo Scientific, Fremont, CA, USA), PR (clone PgR, 1∶50; DAKO), AR (clone AR441, 1∶50; DAKO), or Ki-67 (clone MIB-1, 1∶150; DAKO), immunodetection was performed with biotinylated anti-mouse immunoglobulin, followed by peroxidase-labeled streptavidin using a labeled streptavidin biotin kit with 3,3′-diaminobenzidine chromogen as substrate. Slides were counterstained with Harris hematoxylin. HER2 staining was scored as 0 to 3+, according to the guidelines of the American Society of Clinical Oncology/College of American Pathologists [24]. Cases scored as 3+ were counted as HER2-positive, whereas cases scored as 0 to 1+ were counted as negative. Borderline cases (2+) required further investigation using FISH to measure gene amplification. In FISH analysis, a HER2 gene/chromosome 17 (HER2/Chr17) ratio of higher than 2.2 was considered HER2-positive. ER- and PR-positivity were defined as higher than 10 fmol/mg cytosolic protein or as 10% or higher nuclear IHC staining. AR positivity was defined as>10% tumor or stromal cell positive staining. IHC staining for Ki-67 was scored by counting the number of positively stained nuclei, and expressed as a percentage of total tumor cells.

### Data and statistical analysis

Clinicopathologic data were collected through review of medical records. Clinicopathologic variables included size of aspirated ALNs, tumor size of primary breast cancer, status of HER2, ER, PR, AR, and Ki-67 expression in primary breast cancer, and presence of distant metastasis at the time of diagnosis. Size of aspirated ALNs was measured as the maximum diameter on either transverse or longitudinal scan of preoperative US. Tumor size of primary breast cancers was based on patients' final pathologic reports (113 patients who underwent operation without NAC or preoperative US features obtained before treatment (31 patients who underwent operation after NAC and 20 patients who had not been operated on due to distant metastasis). Information on expression status of HER2, ER, PR, AR and Ki-67 were based on patients' final pathologic reports. For statistical analysis, primary breast cancers were grouped by Ki-67 index (low, <14% or high, ≥14%) and FISH HER2/Chr17 ratio (low, 2.2–4.0 or high, >4.0).

FNA-HER2s of ALNs were compared according to cytologic/final diagnosis using Student's *t*-test. Receiver operating characteristics (ROC) curve analysis was performed to evaluate the diagnostic ability to differentiate benign from metastatic ALNs. The optimal cut-off value was chosen to maximize the sum of sensitivity and specificity. In addition, FNA-HER2s of metastatic ALNs were compared with respect to clinicopathologic variables using Student's *t*-test and ANOVA. The FNA-HER2 statuses of ALNs were divided into two arbitrary categories (negative or positive) according to the FNA-HER2 cut-off value based on ROC analysis. A chi-square test was used for comparison of the FNA-HER2 status of metastatic ALNs to the HER2 status of the corresponding primary breast cancer tissue. Statistical analysis was performed with SAS for Windows, v. 9.0 (SAS Institute, Cary, NC, USA). *P* values less than 0.05 were considered significant.

## Results

### Clinicopathologic patient characteristics

The mean age of the patients was 47.8 years (range, 27–76 years). The mean tumor size of primary breast cancers was 30.2 mm, and the most common histologic type was invasive ductal carcinoma. Status of HER2, ER, PR, AR, and Ki-67 expression for primary breast cancer, and the presence of distant metastasis at the time of diagnosis are presented in Table 1. Of the 165 invasive breast cancers, 59 were HER2-positive (35.8%). Lymph node metastasis was observed in 138 aspirated ALNs from 136 cancers (82.4%). Distant metastasis was observed in 23 cancers (14.0%) from 21 patients, and the metastatic sites consisted of lung, bone, liver, neck node, and brain, in order of frequency.

**Table 1:** Characteristics of 165 invasive breast cancers in 164 patients examined by preoperative axillary US.

Characteristics		Number of primary breast cancers (%)
Histologic type	Invasive ductal carcinoma	148 (89.8)
	Invasive papillary carcinoma	5 (3.0)
	Invasive lobular carcinoma	4 (2.4)
	Mucinous carcinoma	3 (1.8)
	Metaplastic carcinoma	3 (1.8)
	Medullary carcinoma	1 (0.6)
	Neuroendocrine carcinoma	1 (0.6)
HER2	Negative	106 (64.2)
	Positive	59 (35.8)
ER	Negative	69 (41.8)
	Positive	96 (58.8)
PR	Negative	94 (57.0)
	Positive	71 (43.0)
Ki-67	Low	51 (30.9)
	High	84 (50.9)
	N/A	30 (18.2)
AR	Negative	51 (30.9)
	Positive	84 (50.9)
	N/A	30 (18.2)
Lymph node metastasis	Negative	29 (17.6)
	Positive	136 (82.4)
Distant metastasis	Negative	142 (86.0)
	Positive	23 (14.0)

N/A: not available.

### Diagnostic performance of FNA-HER2 in prediction of ALN metastasis

**Figure 1:**
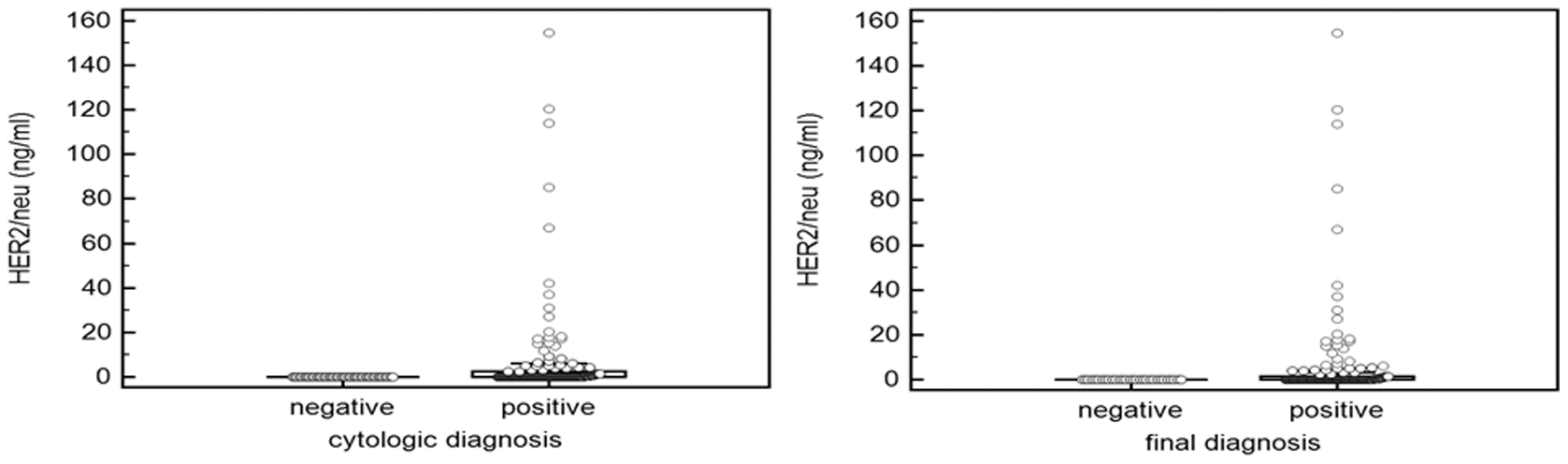
FNA-HER2 of 167 axillary lymph nodes in 165 invasive breast cancers.

Of the 167 ALNs, 138 (82.6%) were metastatic and 29 (17.4%) were benign according to reference standards. The 138 metastatic nodes were confirmed by surgery (n = 116) or with both clinical evidence and cytologic results (n = 22), due to the presence of distant metastasis. The cytology results from US-FNA of ALNs in final metastatic nodes were: metastatic in 125 (74.9%), and benign in 13 nodes (11 benign and 2 insufficient). All 29 of the finally benign nodes had benign cytologic results (27 benign and 2 insufficient material for diagnosis). All 13 of the metastatic ALNs yielding negative cytologic results had negative results (0.00 ng/mL) by FNA-HER2 analysis. The 29 benign ALNs also had no measurable value of HER2. Of the 138 metastatic ALNs, 52 ALNs (37.7%) had positive results (>0.00 ng/mL) by FNA-HER2 analysis. The mean FNA-HER2 (mean 6.31, range 0–154.60) of the metastatic ALNs was higher than that of the benign ALNs, although a substantial number of metastatic ALNs (86/138) had negative results by FNA-HER2 analysis (Figure 1). The area under the ROC curves (Az value) of FNA-HER2s of ALNs was 0.679 for the diagnosis of ALN metastasis, and the optimal cut-off level was 0.00 ng/mL (sensitivity 37.7%, specificity 100%), while the Az value of US-FNA cytology was 0.953 (sensitivity 90.6%, specificity 100%). Addition of FNA-HER2 to the US-FNA cytologic results did not improve the Az value for the prediction of ALN metastasis. In subgroup ROC analysis of 59 patients with HER2-positive primary breast cancers, the Az value of FNA-HER2 was 0.865, and the optimal cut-off level was 0.00 ng/mL (sensitivity 73.1%, specificity 100%) for the diagnosis of ALN metastasis. In this subgroup, the Az value of US-FNA cytology (0.981) was also significantly higher than that of FNA-HER2 (*P* = 0.04).

### Comparison of FNA-HER2 status of metastatic ALNs and HER2 status of their corresponding primary breast cancers

Table 2 shows the concordance and discordance between the FNA-HER2 statuses of metastatic ALNs and the HER2 statuses of the corresponding primary breast cancer tissues in 133 patients with 136 metastatic ALNs. Two metastatic ALNs with insufficient cytologic results were excluded from this analysis. The FNA-HER2 status of ALNs was based on the level of FNA-HER2, and the HER2 status of primary breast cancer tissue was based on a combination of IHC and FISH results. The FNA-HER2 statuses of 108 metastatic ALNs (79.4%) were concordant with the HER2 statuses of their corresponding primary breast cancers. For the concordant metastatic ALNs, the mean FNA-HER2 was 23.1 ng/ml (range 0.2–154.6). There were also 28 metastatic ALNs with discordance (20.6%) between the FNA-HER2 status of the ALN and the HER2 status of the corresponding primary breast cancer tissue. There were 14 metastatic ALNs which were FNA-HER2-positive whose corresponding primary breast cancers were HER2-negative, and the mean FNA-HER2 of these ALNs was 3.0 ng/ml (range 0.1–20.3 ng/ml). Of those, two metastatic ALNs had higher FNA-HER2s (range 6.5–20.3 ng/ml) than did the remainder (range 0.1–4.9 ng/ml). Of these two HER2-negative primary cancers whose corresponding metastatic ALNs had the highest FNA-HER2s, one had an IHC score of 1+ but high gene amplification by FISH, and the other had an IHC score of 1+ before NAC, but an IHC score of 3+ after NAC. There were 14 other metastatic ALNs which were FNA-HER2-negative, but whose corresponding primary breast cancers were HER2-positive.

**Table 2:** Comparison of FNA-HER2 status of 136 metastatic ALNs with HER2 status of the corresponding primary breast cancer tissue.

		FNA-HER2 status of ALNs		
		Positive	Negative	P
HER2 status of primary breast cancer	Positive (n = 52)	38 (73.1)	14 (26.9)	<0.01
	Negative (n = 84)	14 (16.7)	70 (83.3)	

Non-numeric data are presented as number of lymph nodes (percentage).

### FNA-HER2 of metastatic ALNs according to clinicopathologic features

As shown in Table 3, the mean FNA-HER2 of metastatic ALNs was found to be significantly associated with HER2-positivity, high HER2 IHC grade, and high HER2 amplification by FISH of the corresponding primary breast cancers. Additionally, metastatic ALNs from primary ER-negative and PR-negative breast cancers had significantly higher FNA-HER2s than did those that were not ER- and PR-negative. However, there were no significant differences in the mean FNA-HER2s of metastatic ALNs according to metastatic ALN size or the Ki-67 or AR status of the corresponding primary breast cancers.

**Table 3:** Associations between FNA-HER2 of 136 metastatic ALNs and clinicopathologic features of their primary breast cancers.

			FNA-HER2 level (ng/ml)	
Characteristics		Number of axillary lymph nodes	Mean (SD)	*P*
Metastatic ALN size	≤10 mm	30	3.77 (12.89)	0.16
	>10 and ≤20 mm	83	5.69 (17.38)	
	>20	23	14.63 (21.88)	
Primary cancer size^a^	≤20 mm	21	1.68 (3.93)	0.36
	>20 and ≤50 mm	88	7.78 (23.59)	
	>50 mm	15	2.47 (9.52)	
HER2 IHC score	0 or 1	44	0.61 (3.15)	<0.01
	2+	58	4.19 (16.32)	
	3+	33	19.71 (35.95)	
HER2 amplification by FISH^a^^,^ b	None	68	0.31 (1.09)	<0.01
	Low	18	26.77 (45.52)	
	High	29	12.67 (24.51)	
HER2 status (IHC+FISH)	Negative	84	0.50 (2.40)	<0.01
	Positive	52	16.91 (32.98)	
ER	Negative	57	13.03 (30.50)	<0.01
	Positive	79	2.26 (10.53)	
PR	Negative	80	10.01 (26.45)	0.04
	Positive	56	2.15 (11.52)	
Ki-67^a^	Negative	45	4.48 (11.73)	0.29
	Positive	62	9.55 (30.01)	
AR^a^	Negative	43	7.52 (22.10)	0.97
	Positive	64	7.35 (25.56)	
Distant metastasis	Negative	113	6.24 (20.28)	0.53
	Positive	23	9.40 (28.85)	

IHC: immunohistochemistry.

a Cases with no available results for each characteristic were excluded from data analysis: two cases for primary cancer size, 22 cases for HER2 FISH, and 29 cases for AR and KI-67 status.

b HER2 FISH test were performed for 58 cases with borderline (2+) IHC results and for 57 cases on the recommendation of either the physician or the pathologist.

In addition, we analyzed the clinicopathologic features of 52 HER2-positive primary breast cancers according to the FNA-HER2 status of their metastatic ALNs (Table 4). In this subgroup analysis of HER2-positive cancers with ALN metastasis, distant metastasis was more frequently seen in the discordant group (HER2-positive primary cancer but FNA-HER2-negative metastatic ALN) than in the concordant group, with statistical significance (*P* = 0.04). The mean size of metastatic ALNs and primary cancers, HER2 amplification by FISH, and the HER2 IHC grade of primary breast cancers were not significantly different between the two groups.

**Table 4:** Analysis of clinicopathologic features of 52 HER2-positive primary breast cancers according to FNA-HER2 status of their metastatic ALNs.

	FNA-HER2 status		
Characteristics	Positive (concordant)	Negative (discordant)	*P*
Number of cancers	38	14	
Mean size of metastatic ALNs (mm)	15.2±8.5	13.2±6.3	0.44
Mean size of primary cancers (mm)*	29.5±15.1	26.8±13.4	0.61
HER2 status (IHC+FISH)*			0.59
IHC 2+ and low HER2 amplification by FISH	7 (18.4)	1 (7.2)	
IHC 2+ and high HER2 amplification by FISH	8 (21.1)	3 (21.4)	
IHC 3+	23 (60.5)	10 (71.4)	
Distant metastasis			0.04
Negative	34 (89.5)	9 (64.3)	
Positive	4 (10.5)	5 (35.7)	

Numeric data are presented as mean ± SD (standard deviation).

Non-numeric data are presented as number of cancers (percentage).

*Cases with no available results for each characteristic were excluded from data analysis: two cases for primary cancer size, and 5 cases for HER2 FISH.

## Discussion

US-FNA is a commonly used diagnostic method for preoperative staging of ALNs. However, a substantial number of false negative results can occur in US-FNA cytology [10], and therefore several markers in US-FNA specimens, such as CEA, CA15-3, and CYFRA 21-1, have been investigated in previous studies to improve the diagnostic performance of preoperative US-FNA cytology [12], [13]. In the current study, we evaluated whether FNA-HER2 of ALN can improve the diagnostic performance of US-FNA cytology in preoperative diagnosis of ALN metastasis. Our results showed that FNA-HER2 did not improve the sensitivity of US-FNA cytology, because a substantial number of metastatic ALNs (62.3%), including metastatic ALNs with false negative cytologic results, gave negative results on FNA-HER2. This may be a predictable result, because HER2 expression is seen in some portion of overall breast cancers. Therefore, FNA-HER2 may not be useful in preoperative ALN staging for overall breast cancer patients. However, all ALNs with positive FNA-HER2 were metastatic; moreover, a higher FNA-HER2 in our metastatic ALNs was significantly associated with HER2-positivity of corresponding primary breast cancers in this study (*P*<0.001). Accordingly, the Az value of FNA-HER2 was higher in a subgroup analysis of HER2-positive breast cancers (0.865), than for overall patients (0.679) in this study. Nevertheless, the Az value of FNA cytology (0.981) was still higher than that of FNA-HER2 in this subgroup, and all 13 metastatic ALNs yielding negative cytologic results had negative FNA-HER2 (0.00 ng/mL). Considering these findings, FNA-HER2 of ALNs may not improve the diagnostic performance of FNA cytology in preoperative diagnosis of ALN metastasis in breast cancer patients.

Since the clinical application of the humanized anti-HER2 antibody, trastuzumab, as a clinical anticancer agent for HER2-positive breast cancer, selection of trastuzumab treatment has been based on the HER2 status of primary tumors [16], [25], [26]. This is due to initial studies which suggested that the HER2-status of breast cancer is stable through the course of the disease [27], [28]. However, some researchers have reported that the HER2 status of primary tumors can be discordant with the HER2 status of metastatic lymph nodes [29]–[31]. This HER2 status discrepancy between primary breast cancer and metastatic lymph node suggests that patients who have HER2-negative primary cancers but HER2-positive metastatic lymph nodes may miss the opportunity for successful treatment with tratsuzumab combination therapy. Nonetheless, the majority of breast cancer patients have concordant HER2 statuses between their primary cancer and metastatic ALN [29]–[31]. It would be clinically inefficient to evaluate the HER2 status of both the primary cancer and metastatic ALN using IHC/FISH analysis, which requires the time-consuming process of slide preparation, and is mainly applicable to tissue samples obtained by surgical or core needle biopsy [32]. Accordingly, we hoped that FNA-HER2 of ALN might be a clinically useful method for preoperative detection of metastatic ALNs with HER2 discrepancy, given that US-FNA is a widely used method for the preoperative diagnosis of ALN metastasis. In this study, the majority (79.4%, 108/136) of the FNA-HER2 statuses of metastatic ALNs were concordant with the HER2 statuses of their corresponding primary cancers as determined by IHC/FISH. On the other hand, discordant FNA-HER2 statuses of metastatic ALNs were found in 20.6% (28/136) of metastatic ALNs, which is consistent with values (16–34%) reported in previous studies that used IHC/FISH analysis for the evaluation of the HER2 statuses of primary and metastatic tumors [29]–[31]. There are several hypotheses that may account for the discordance between the FNA-HER2 of metastatic ALNs and the IHC/FISH HER2 status of primary breast cancers. First, it may be attributed to the genetic mutations in HER2 that can occur during tumor progression [33]. On the other hand, there can be small neoplastic clones that not only may have a different HER2 status from the majority of the primary cancer cells, but also may have enhanced potential to metastasize, leading to metastases of clones with discordant HER2 genes from the primary tumor [34], [35]. Our one discordant case, which was designated as HER2-negative primary breast cancer with FNA-HER2 positive metastatic ALN before NAC, but as HER2-positive breast cancer after NAC, may support this hypothesis. However, most of our discordant cases underwent NAC and exhibited pathologic complete response in surgical specimens, and thus we could not compare the pre- and post-treatment HER2 statuses of these primary cancers. Consequently, it may be inferred that the FNA-HER2 of metastatic ALNs may identify metastatic ALNs exhibiting HER2 discrepancy with the primary tumor. In addition, FNA-HER2 analysis of ALNs may have a potential role in decision-making with respect to the use of trastuzumab treatment and the development of targeted therapy for breast cancer patients.

We evaluated the association between clinicopathologic features of primary breast cancers and the FNA-HER2 of their metastatic ALNs. FNA-HER2 levels of metastatic ALNs were positively associated with HER2-positivity, high HER2 IHC score, and high HER2 amplification by FISH, of primary breast cancers. We also observed that metastatic ALNs of ER/PR-negative breast cancers showed higher FNA-HER2s than those that were not ER/PR-negative. However, this finding may be a consequence of the fact that the majority of our ER/PR-negative breast cancers with ALN metastasis were HER2-positive. In a subgroup analysis of HER2-positive breast cancers with ALN metastasis, we found that distant metastasis was more frequently seen in the FNA-HER2 negative group than in the FNA-HER2 positive group, which was statistically significant. A recent study has shown that breast cancer patients with HER2 discordance between primary and metastatic tumors had worse overall survival and post-recurrence survival compared to the concordant group [36]. Considering this with our own results, it may be inferred that patients with HER2-positive breast cancer may have poor prognoses in the case of HER2-discordant ALN metastasis, relative to those with HER2-concordant ALN metastasis. This may be explained by intratumoral heterogeneity in the HER2 expression of cancer cells, which was significantly associated with decreased disease-free survival of HER2-positive breast cancer patients in a recent study [37]. Therefore, further studies with larger numbers of patients may help confirm the relationship between HER2 discrepancy between primary cancer and metastatic ALN, and poor prognosis.

This study has several limitations. First, our sample size was relatively small. Second, we measured FNA-HER2 of ALNs by ELISA targeting the ECD of the HER2 receptor. Secretion of the ECD of the HER2 receptor may be affected by tumor load/size, or by activity of tissue metalloproteinase [38]. Therefore, a small number of cancer cells or a difference in the activity of tissue metalloproteinase in metastatic ALNs may result in variability in our FNA-HER2 measurements, which could lead to a FNA-HER2 discordance with the HER2 status of the corresponding primary breast cancer. Therefore, further studies with larger numbers of patients are necessary for validation of our FNA-HER2 analysis. Third, we only included ALNs with suspicious US features, because US-FNA was not routinely recommended for ALNs without suspicious features at our institution. Finally, we did not analyze the cost effectiveness of FNA-HER2 analysis of ALN.

## Conclusions

FNA-HER2 positive metastatic ALNs were significantly associated with HER2-positivity of primary breast cancers, although FNA-HER2 of ALNs did not improve the diagnostic performance of FNA cytology with respect to preoperative diagnosis of ALN metastasis of overall patients. In addition, FNA-HER2 analysis of ALN may help to develop more personalized treatment protocols for breast cancer patients by determining the concordance or discordance of HER2 status between primary cancers and metastatic ALNs.
